# Sample preparation techniques for extraction of vitamin D metabolites from non-conventional biological sample matrices prior to LC–MS/MS analysis

**DOI:** 10.1007/s00216-022-04097-1

**Published:** 2022-05-02

**Authors:** Anastasia Alexandridou, Dietrich A. Volmer

**Affiliations:** Bioanalytical Chemistry, Humboldt University Berlin, Brook-Taylor-Str. 2, 12489 Berlin, Germany

**Keywords:** Sample preparation, Alternative/non-conventional biological samples, Vitamin D metabolites, 25-Hydroxyvitamin D_3_, LC–MS/MS

## Abstract

The determination of vitamin D metabolites as status marker or for diagnostic purposes is almost entirely conducted from blood serum or plasma. Other biological matrices, however, have also interested researchers, for two main reasons: (1) alternative matrices may allow non-invasive sampling, permit easier sample transfer and require less demanding storage conditions; and (2) the levels of vitamin D metabolites in other body compartments may further aid the understanding of vitamin D metabolism and function. Thus, the development of reliable and efficient sample preparation protocols for sample matrices other than serum/plasma, which will remove potential interferences and selectively extract the targeted metabolites, is of great importance. This review summarizes sample preparation methods for measurement of vitamin D metabolites using liquid chromatography-(tandem)mass spectrometry in more than ten different human tissues, including hair, saliva, adipose tissue, brain and others.

**Graphical abstract:**

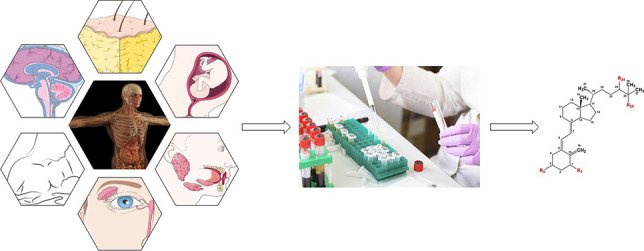

## Introduction

Vitamin D deficiency is a global phenomenon, present in all age groups, necessitating reliable and accurate high-throughput vitamin D status measurement assays. The measurement of blood serum/plasma concentrations of different vitamin D metabolites is also widely used for clinical assessment and following the progression of several diseases [1]. However, analysis of vitamin D metabolites in biological sample matrices other than blood could potentially provide complementary or even additional information to further understand vitamin D metabolism and function. Liquid chromatography (LC), and to a lesser extent gas chromatography (GC), and mass spectrometry (MS) are the preferred analytical techniques for simultaneous measurement of multiple vitamin D metabolites, as reviewed in a number of excellent papers [2–7].

LC–MS/MS is often described as the ‘gold standard’ method for vitamin D analysis as it provides increased sensitivity and specificity over other assays [4] as well as the ability to analyse multiple metabolites simultaneously [8]; however, it often requires trained laboratory personnel. Immunoassays dominate 25(OH)D determination in clinical laboratories [6] because they are fully automated and offer much faster measurements of 25(OH)D levels than LC–MS/MS methods. Moreover, sample preparation is usually less complicated for immunoassays than for LC–MS/MS. However, immunoassays can suffer from cross reactivity between different vitamin D species, providing unreliable results due to poor antibody specificity [5]. Also, most of the commercially available immunoassays cannot differentiate vitamin D epimers or 25(OH)D_3_ from 25(OH)D_2_ [3].

Sample preparation protocols for vitamin D determination are usually applied to blood serum or plasma samples. The most common steps include protein precipitation, extraction and sometimes derivatization as final step. Protein precipitation will remove the proteins and release vitamin D species bound to proteins. Extraction separates the analytes of interest from other co-existing endogenous and exogenous substances and often also achieves preconcentration. Different types of extraction procedures have been developed such as liquid–liquid extraction (LLE) [9–14], solid-phase extraction (SPE) [15–17] and supported liquid extraction (SLE) [18–22]. Derivatization usually enhances the ionization efficiency for specific compounds resulting in increased sensitivity of the method and providing the opportunity for low abundant metabolites to be measured. Derivatization can sometimes also provide enhanced specificity. It does, however, increase the sample preparation time and thus total time of analysis as well as total cost. Vitamin D metabolites offer two possibilities for chemical derivatization: (1) the *cis* diene moiety at C-5/6 and C-19 and (2) the hydroxyl groups of the metabolites. As chemical derivatization is outside the scope of this review, the present authors refer interested readers to a comprehensive summary of the available derivatization techniques for vitamin D metabolites [7].

Several additional factors may also influence the decision to switch to an alternative sample type other than blood, such as the: (1) the limited availability of samples (e.g. post-mortem cases), (2) problems within the sampling group (e.g. challenges in obtaining blood samples from new-borns or from subjects with damaged veins such as elderly or chemotherapy patients), (3) the sampling invasiveness, (4) the analytes’ stability, (5) transport/storage conditions of the samples, (6) the possibility of contamination and (7) the possibility of replacement or adulteration of samples (e.g. doping analysis).

Every biological matrix comes with a unique set of advantages, limitations and challenges. Endogenous and exogenous compounds in the sample have the potential to cause interferences and influence the accuracy of the analysis. To overcome this problem, it is necessary to develop sample preparation techniques that allow selective and efficient isolation of the target analytes from the matrix. Next, a suitable combination of sample preparation and subsequent separation and detection method must be achieved [23]. Moreover, preconcentration is often the goal if the levels of the compounds of interest in the specific matrix are very low. Sometimes, the final step of sample pre-treatment is derivatization of the analytes.

This review describes the utility of different biological sample matrices beyond serum/plasma for determination of vitamin D metabolites. Previous reviews have focused mainly on human body fluids [24–26] or food [27]. The present review summarizes the sample preparation steps prior to LC–MS/MS analysis and emphasizes the challenges and the critical factors that should be considered before developing a sample pre-treatment protocol.

## Human biological specimens for evaluation of vitamin D

As most biological specimens contain a large number of endogenous and exogenous compounds, which could possibly interfere with the analysis of vitamin D metabolites, separation of interferences, purification, preconcentration and sometimes chemical modification has to be performed prior to instrumental analysis [28].

The possible human biological materials of interest include biological fluids (blood, serum, plasma, urine, sweat, saliva, cerebrospinal fluid, aqueous humour, amniotic fluid, breast milk, tears), organs, tissues, bones, teeth, embryos, skin, nail clippings, earwax, cells, gametes, hair, proteins, DNA, RNA, faeces, meconium, gastric content, exhaled air and others. Importantly, not all of these materials have been investigated for the levels of vitamin D metabolites. Organisms that are isolated from human samples, such as bacteria or viruses, are not considered human biological specimens in this review.

The most important biological matrices for clinical evaluation of vitamin D metabolites are blood (and blood-derived products) and urine, which we consider ‘conventional’ matrices in this review. All other, more ‘exotic’ matrices could potentially supply complementary information concerning the metabolism and catabolism of vitamin D, may reveal exposure over longer time windows than blood or urine or are simply easier to obtain or to transport, e.g. hair and nail clippings. The latter reason is particularly useful in population-based studies (including subjects in remote locations) in certain subject groups such as the elderly or new-borns [29], or even in clinical trials [30], where hair samples can be used to monitor adherence over longer periods.

In the following sections, the present authors will go through human specimens where vitamin D metabolites have been detected and will try to underline their general usefulness and the information that may be obtained from these materials. We aim to answer the following questions for each of these matrices: is the information obtained from the non-conventional specimens as complete as that from the conventional material? Does it provide complementary information? Are there any parameters to be taken into consideration before choosing the biological material prior to chemical analysis? Should non-conventional biological samples, which most of the time require more complicated sample preparation method than conventional blood samples, have a place in routine clinical analysis?

## Sample preparation of alternative/non-conventional biological samples prior to chromatographic separation and mass spectrometry analysis

Sample pre-treatment for vitamin D can be challenging due to the unstable nature of the compound and its metabolites under certain conditions, e.g. storage conditions (light, oxygen, temperature). For example, ‘back-isomerization’ can occur as a result of heat exposure [31]. Additionally, interferences from endogenous and exogenous compounds should always be expected. Finally, the extremely low concentrations of some of the metabolites in some matrices are a major problem requiring preconcentration or chemical derivatization. Standard sample preparation steps include extraction of the analytes, purification, preconcentration and/or derivatization. While derivatization is often used to improve accuracy, sensitivity and reproducibility, it can further contribute to the existing analytical problems with reagent interferences, derivative instability, inability to derivatize certain functional groups and longer analysis times. The nature of the sample matrix and the following ionization technique are some of the important factors that will determine the steps of the sample pre-treatment.

**Fig. 1:**
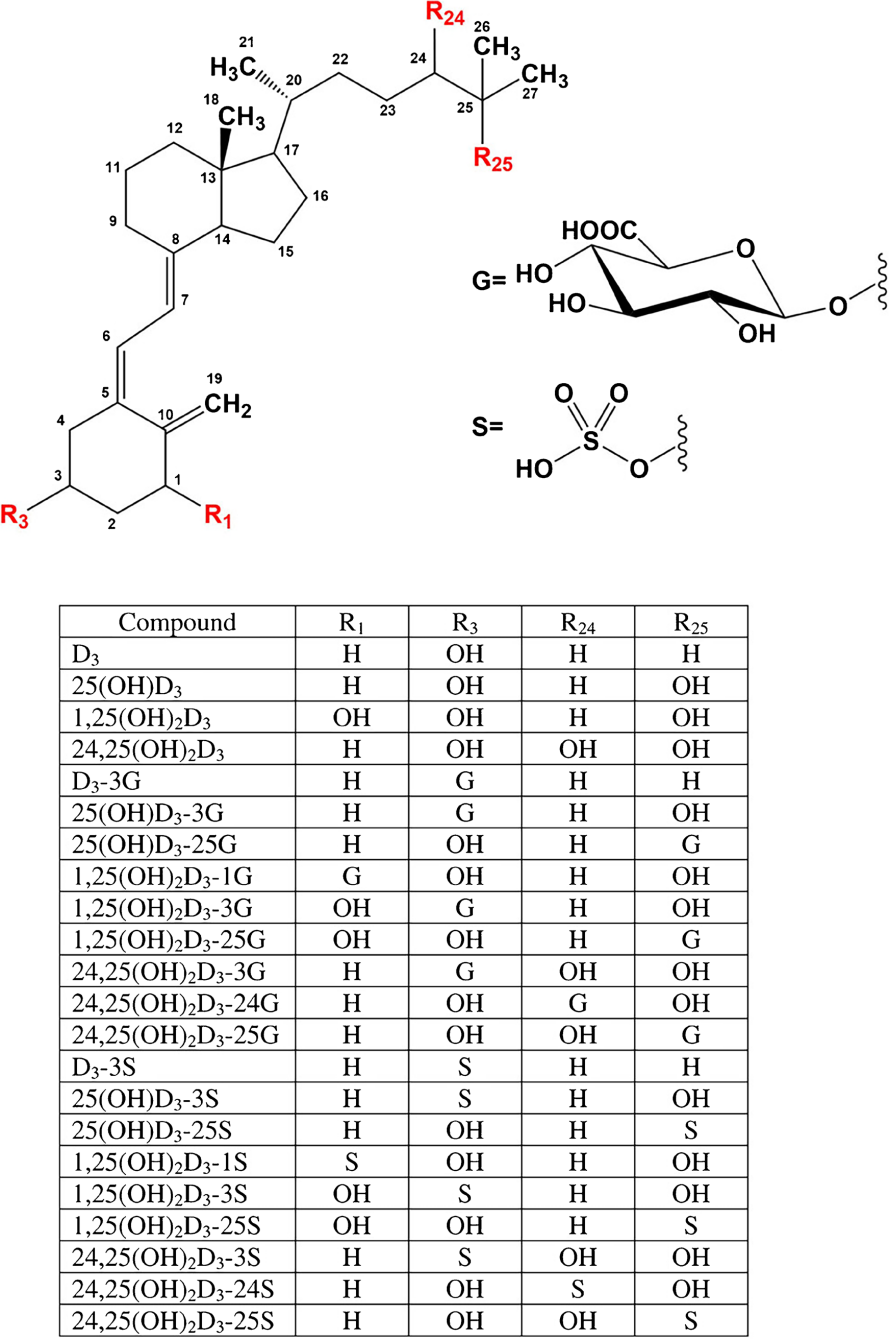
Chemical structure of vitamin D_3_ metabolites. Reprinted from ref. [7] with permission under the CC BY 4.0 license

This section will focus on sample preparation of biological samples beyond blood (and blood-derived matrices) for components of the vitamin D metabolic cascade. Figure 1 summarizes the most important vitamin D_3_ metabolites described in the following sections [7]. More details on the metabolic cascade can be found elsewhere, e.g. [32, 33], as well as detailed information on sample preparation for conventional blood-related samples or for food matrices [26, 27].

### Adipose tissue

Adipose tissue plays a crucial role in steroid hormone synthesis and metabolism [34]. It is believed to be storage reservoir of vitamin D, and for that reason, it is relevant for vitamin D research [35]. Obese individuals have exhibited significant lower circulating concentrations of 25(OH)D than non-obese subjects [34]. There are two hypotheses for this observation: (1) fat tissue is responsible for increased vitamin D sequestration, so higher amounts of adipose tissue can lead to increased metabolic clearance [34]. (2) The bioavailability of vitamin D decreases because of its storage in fat tissue [36]. It is likely that vitamin D that is diffusing into fat cells originates from the free vitamin D fraction, as the circulating levels are bound to vitamin D-binding protein (DBP) and albumin [37].

Blum et al. analysed subcutaneous fat tissue using a liquid chromatography-atmospheric pressure chemical ionization-mass spectrometry (LC-APCI-MS) method and quantified vitamin D_3_ in 17 obese men and women [38], with a mean concentration of 102.8 nmol/kg. Samples were obtained from the subjects after gastric bypass surgery and 0.2–0.25 g of tissue was used for each analysis. The first step of the sample preparation was homogenization of the sample using 2.6 mL of a 1:1 (v/v) H_2_O:ethanol mixture using a homogenizer. Subsequently, 200 μL of the homogenized tissue was transferred to a glass tube and deuterated vitamin D_3_ internal standard was added, followed by evaporation under a N_2_ stream at 45 °C; 1 mL of 30% KOH was added to the dry residue and the sample was shaken (room temperature, 250 rpm, 3 h). LLE was chosen for further sample preparation: 2 mL of H_2_O, 3 mL of hexane and 3 mL of ethanol were added and the tube was vortexed (5 min), followed by centrifugation at 4 °C (3,000 rpm, 5 min) for optimal results. SPE using a C-18 column was applied as a last step of purification prior to injection onto the LC column.

Martinaityte et al. described a slightly different approach to quantify 25(OH)D_3_ and vitamin D_3_ [39, 40]. Briefly, the internal standards, 0.2 g of sodium ascorbate, 3 mL of 60% KOH and 9 mL of ethanol were added to 0.2–1 g of adipose tissue. The samples were left overnight saponification (16–18 h, room temperature), followed by LLE (13 mL H_2_O, 10 mL of 20% ethyl acetate in heptane, 1 min). The aqueous phase was re-extracted twice and the organic phases were combined and evaporated to dryness, followed by SPE (silica) as final step.

The main difference of Martinaityte et al.’s assay to Blum et al.’s method was the addition of a subsequent derivatization step using 4-phenyl-1, 2, 4-triazoline-3,5-dione (PTAD), which enabled very sensitive analysis with limits of quantification of 0.1 ng/g. This came at the expense of analysis time; however, Blum et al.’s method was faster not only because no time-consuming derivatization was performed but also because the saponification step was conducted faster, with smaller amounts of tissue sample needed.

Subsequently, Didriksen et al. followed the same sample preparation and derivatization protocol as Martinaityte et al. to analyse subcutaneous fat tissue [37].

In conclusion, sample pre-treatment of adipose tissue can be time consuming and includes multiple steps, mainly (1) saponification, (2) LLE and (3) SPE. The reported difficulties in the studies described above underlined the complexity of adipose tissue as sample matrix.

**Fig. 2:**
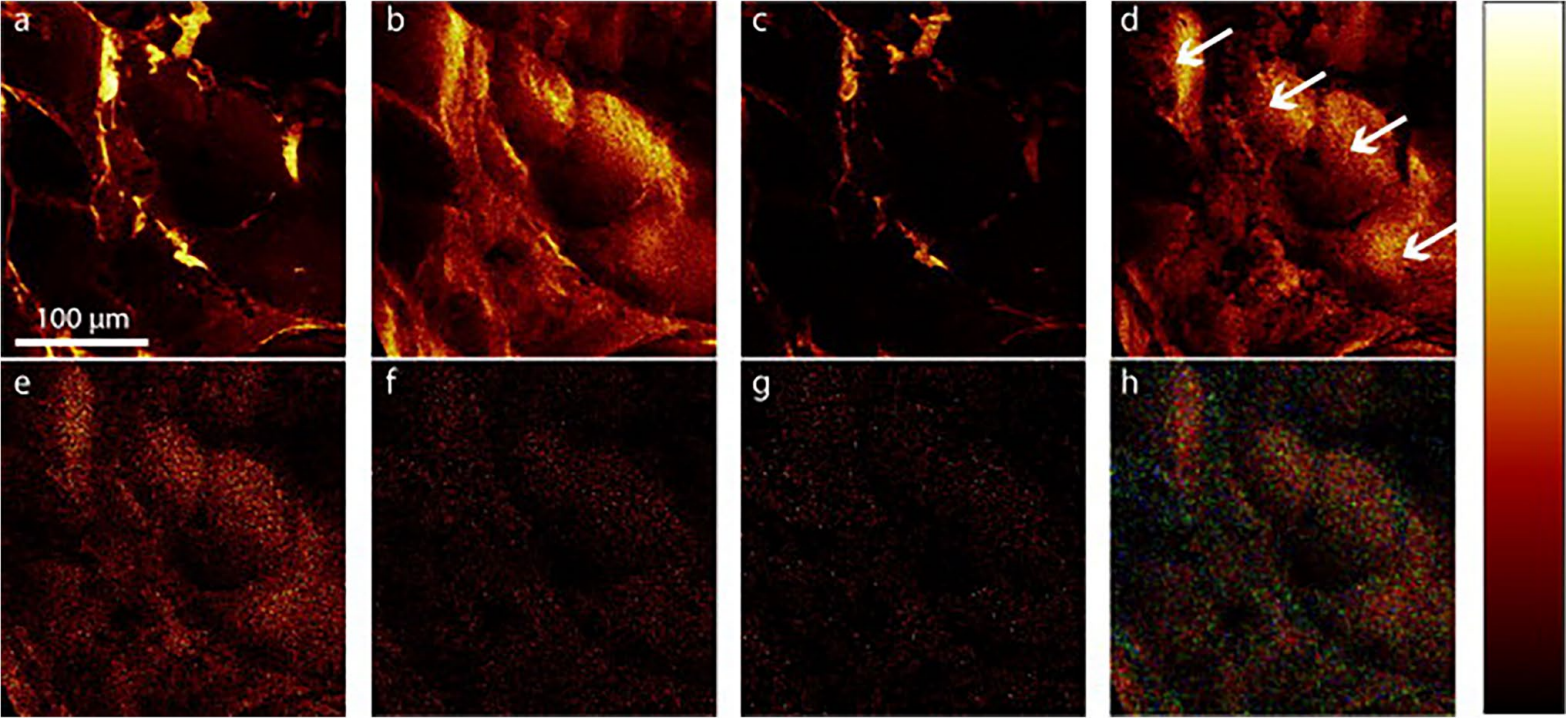
TOF–SIMS images of high-pressure-frozen, freeze-fractured visceral adipose tissue biopsies of 258 × 258 μm^2^ size from one obese individual. The lateral resolution of the images is ~ 1 μm. (**a**–**d**) Distribution of secondary ions of sodium [Na]^+^ (**a**) and potassium [K]^+^ (**b**), the phosphatidylcholine headgroup [C5H15PNO4]^+^ *m/z* 184.2 (**c**) and diacylglycerol (DAG) at *m/z* 551.2 (**d**) (**e**), vitamin D_3_ [M-OH]^+^ at *m/z* 367.1 (**f**), 25(OH)D_3_ [M-OH]^+^ at *m/z* 383.2, (**g**) 1,25(OH)_2_D_3_ [M-OH]^+^ at *m/z* 399.8. (**h**) An overlay image of vitamin D_3_ [M-OH]^+^ in red, 25(OH)D_3_ in green, and 1,25(OH)_2_D_3_ in blue. The adipocytes are indicated by arrows (**d**). All images are presented in the same colour scale as the included colour bar, ranging from black representing 0 ion intensity to white indicating the highest ion signal intensity in the image. All other colours assigned using a linear relationship. Scale bar for all images = 100 μm. Reprinted from ref. [41] with permission from Elsevier

An entirely different approach was reported by Malmberg et al., who localized (Fig. 2) vitamin D_3_, 25(OH)D_3_ and 1,25(OH)_2_D_3_ in the adipocyte using mass spectrometry imaging [41] by time-of-flight secondary ion mass spectrometry (TOF–SIMS). In addition to localization of vitamin D metabolites at a cellular level, this technique enabled semi-quantitative determination of the target molecules in adipose tissue of different groups of individuals. According to the authors, high-pressure freezing was a suitable sample preparation technique as it decreased ion re-localization to a minimum.

### Amniotic fluid

Amniotic fluid provides a physiological buffer and immune protection for the foetus as well as a pool of nutrients [42]. In order to reflect vitamin D status and metabolism in the feto-placental unit, vitamin D metabolites are determined in amniotic fluid. Several studies have indicated that there is independent metabolism of vitamin D in the feto-placental unit [43]. The foetus obtains many nutrients by swallowing amniotic fluid; in fact, it swallows 300–450 mL of amniotic fluid per day [42], which contain up to 200 ng of 25(OH)D, an amount that is considered significant [44].

Lazebnic et al. measured 25(OH)D, 1,25(OH)_2_D and 24,25(OH)_2_D in amniotic fluid samples [43]. LLE was performed from 8 to 25 mL of amniotic fluid and chloroform–methanol (2:1, v/v) after addition of internal standards. The lipids were separated from vitamin D metabolites using a Sephadex LH-20 column and final purification of 25(OH)D and 1,25(OH)_2_D was achieved using a normal-phase HPLC column (μPorasil). For purification of 24, 25(OH)_2_D, a C-18-μBondapak column was used. The final determination of the metabolite concentrations was achieved by using a competitive protein-binding radioassay. Koskinen et al. followed a similar sample preparation protocol to measure 25(OH)D in amniotic fluid using a competitive protein-binding assay [44]. After addition of the internal standard to 7–10 mL of amniotic fluid, the sample was first purified on a C-18 Sep-Pak cartridge, followed by a Nucleosil 5–7 silicic acid column.

**Fig. 3:**
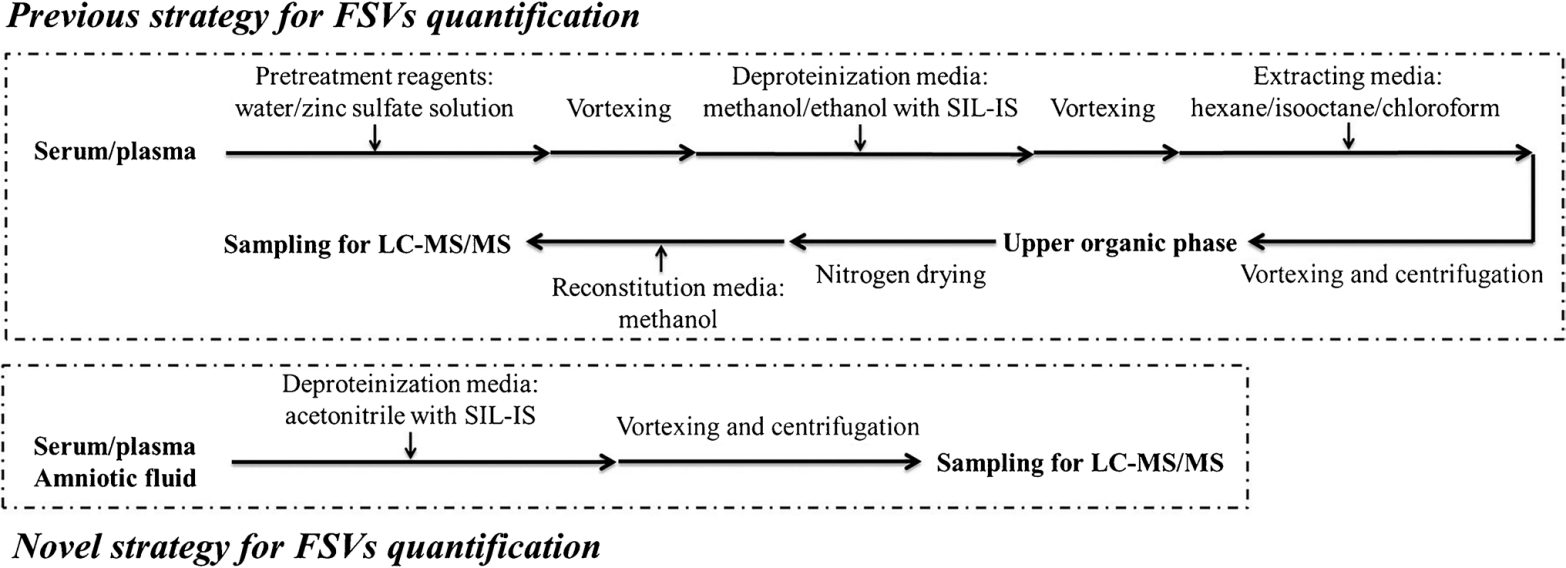
Schematic procedure of previous sample preparation protocols compared to a single-step sample pre-treatment strategy for fat-soluble vitamin (FSV) quantification in plasma and amniotic fluid. Reprinted from ref. [45] with permission under the CC BY 4.0 license

More recently, Le et al. developed a LC–MS/MS method for measuring vitamin D metabolites in human amniotic fluid [45], which, to our knowledge, is the first assay since the 1980s. The method is novel because it permitted (1) the determination of multiple fat-soluble vitamins (A, D and E); (2) the separation of 25(OH)D_2_ and epimers 3α-25(OH)D_3_ and 3β-25(OH)D_3_; (3) the use of matching deuterated internal standards for each vitamin D compound; and (4) the application of a very simple one-step sample pre-treatment procedure (Fig. 3). After collection of the amniotic fluid, the sample was centrifuged and the supernatant was stored at − 80 °C. Acetonitrile (90 μL) was added with the internal standards to 60 μL of amniotic fluid. Protein precipitation was achieved after vortexing and centrifugation at 4 °C; the resulting supernatant was transferred to LC vials for measurement. Le et al.’s protocol is fast and simple, while requiring only very small volume of sample and organic solvent compared to the 1980s approaches, reflecting strong improvements of the analytical instrumentation, in particular LC and MS, in the past 40 years.

### Cerebrospinal fluid (CSF) and human brain

Vitamin D plays a key role in brain development, physiological function [46] and neurogenerative diseases [47]. Vitamin D, 25(OH)D, 1,25(OH)_2_D and 24,25(OH)_2_D can be determined from CSF since they are transferred from plasma into CSF. Passive diffusion, active transport or other mechanisms of transport have been suggested to explain their presence in CSF and brain [48]. Results by Holmøy et al., however, do not support the active transport of 25(OH)D to CSF [49].

Holmøy et al. analysed CSF (400 μL) after addition of internal standard without any prior sample pre-treatment [49]. LC–MS was used for quantification of 25(OH)D. He et al. measured 25(OH)D_3_ and 24,25(OH)_2_D_3_ using LC–MS/MS after PTAD derivatization [46]. The authors used 200 μL of CSF and sample preparation included protein precipitation after addition of acetonitrile and evaporation of the supernatant to dryness prior to derivatization. Moghtaderi et al., Johansson et al. and Lee et al. measured 25(OH)D_3_ in CSF using an enzyme-linked immunoassay (ELISA) electrochemiluminescence assay [50–52]. In these studies, CSF was shown to be relatively easy to handle biological sample matrix, not requiring complicated and time-consuming sample preparation processes.

Fu et al. were the first to analyse vitamin D_3_, 25(OH)D_3_ and 1,25(OH)_2_D_3_ in human brain using LC–ESI–MS/MS [53]; vitamin D_3_ was not detected in the examined brain samples. The purification of brain samples can be very challenging due to the presence of lipids, which comprise approximately 60% of the brain’s dry weight. In Fu et al.’s study, 0.1 g of brain tissue was homogenized in the presence of 0.5 mL of dichloromethane:methanol (1:1, v/v). The internal standards and 0.5 mL of dichloromethane:methanol (1:1, v/v) were added to the homogenized sample. After vortexing and centrifugation, the supernatant was transferred to a new sample tube and a second step of LLE followed after addition of a 0.5-mL methylene chloride:methanol mixture. After vortexing and centrifugation, the second supernatant was combined with the first supernatant and the combined mixture was dried (N_2_, 60 °C), followed by PTAD derivatization.

Other reported sample preparation methods describe animal brain samples. In one study by Ahonen et al., one-quarter of the whole mouse brain was homogenized and extracted by LLE in the presence of 0.5 mL of dichloromethane:methanol (1:1, v/v) in an ice bath (ultrasonication, 1 min) after addition of the internal standards [54]. The extraction was repeated twice. The supernatants resulting from the extraction of the remaining quarters were combined, dried and reconstituted prior to LC injection. Xue et al. used 90 mg of rat brain tissue, which was homogenized in 1 mL of acetonitrile in the dark after addition of internal standard [55]. After vortexing and centrifugation, the acetonitrile was transferred to a new tube, dried under N_2_ gas and derivatized with PTAD prior to analysis.

Fu et al.’s protocol required more sample preparation steps than Ahonen et al.’s method, since it incorporated a derivatization step in addition to two steps of LLE. Fu et al.’s LC–ESI–MS/MS method gave LODs for vitamin D_3_, 25(OH)D_3_ and 1,25(OH)_2_D_3_ in porcine brain of 25, 50 and 25 pg/g, respectively. Xue et al.’s approach was the simplest among the three described methods as it did not require a dedicated LLE step but rather used only protein precipitation and derivatization. The authors reported an analytical response increase of approximately 100 times after PTAD derivatization compared to underivatized analysis. The LC–ESI–MS/MS method achieved a LOQ for 25(OH)D_3_ of 0.10 ng/mL and 0.25 ng/mL for 24,25(OH)_2_D_3_ in rats’ brain. Unfortunately, a direct comparison of the analytical methods is difficult since the chemical nature of the matrix varies between human and animal samples, affecting the matrix background and thus the sensitivity of the method. Also, in these studies, LODs and LOQs are expressed in different units (pg/g versus ng/mL), making direct comparisons impossible.

A very important factor affecting the quality and accuracy of the results, which is often overlooked or not evaluated, is the impact of storage time on the stability of a compound in a specific matrix. Fu et al. examined the influence of freezer storage time on 25(OH)D_3_ levels in human brain tissue for up to 13 years [56] and observed some very interesting results. The authors demonstrated that the same compound will show different stabilities depending on the sample matrix. The concentration levels of 25(OH)D_3_ in brain, which was stored for more than 6 years, were significantly lower than those in brain stored for only 1 year or less. The authors’ conclusions were that brain samples can be stored at − 80 °C for up to 6 years in contrast to serum samples, which can be stored at − 25 °C for up to 24 years [57–59].

### Eye-related biological matrices

Tear film consists mostly of water (~ 98%). Lipids are also present in tears; they decrease friction and reduce evaporation. In addition, both water- and fat-soluble vitamins were found in tears [60]. The function and metabolism of vitamin D in the eye are not fully understood, however, and more research is required [61–63].

Goksugur et al. were the first group to measure 25(OH)D in human tears using ELISA [64]. After sampling by capillary suction, samples were stored at − 80 °C. The interesting observation from that study was that vitamin D levels were higher in tear fluid than in plasma. The authors’ observation is in agreement with results by Sethu et al. and Lai et al., who measured 25(OH)D using ELISA and electrochemiluminescence assay [65, 66]. In contrast, Khaksari et al. observed lower levels of fat-soluble vitamin E in tears than in serum [60, 67]. The authors explained this finding with the limited lipid content of tears, in which the fat-soluble vitamins are dissolved.

Usually, absorbent paper strips are used for tear fluid sampling and sample extraction is performed prior to analysis [60, 65, 66].

Aqueous humour is the clear fluid between the cornea and the front of the vitreous. It is a matrix of potential interest since it is responsible for the transportation of nutrients to the lens and cornea. Vitreous humour is the clear gel that fills the eyeball behind the lens and supports the shape of the eye while it transmits light to the retina [68].

**Fig. 4:**
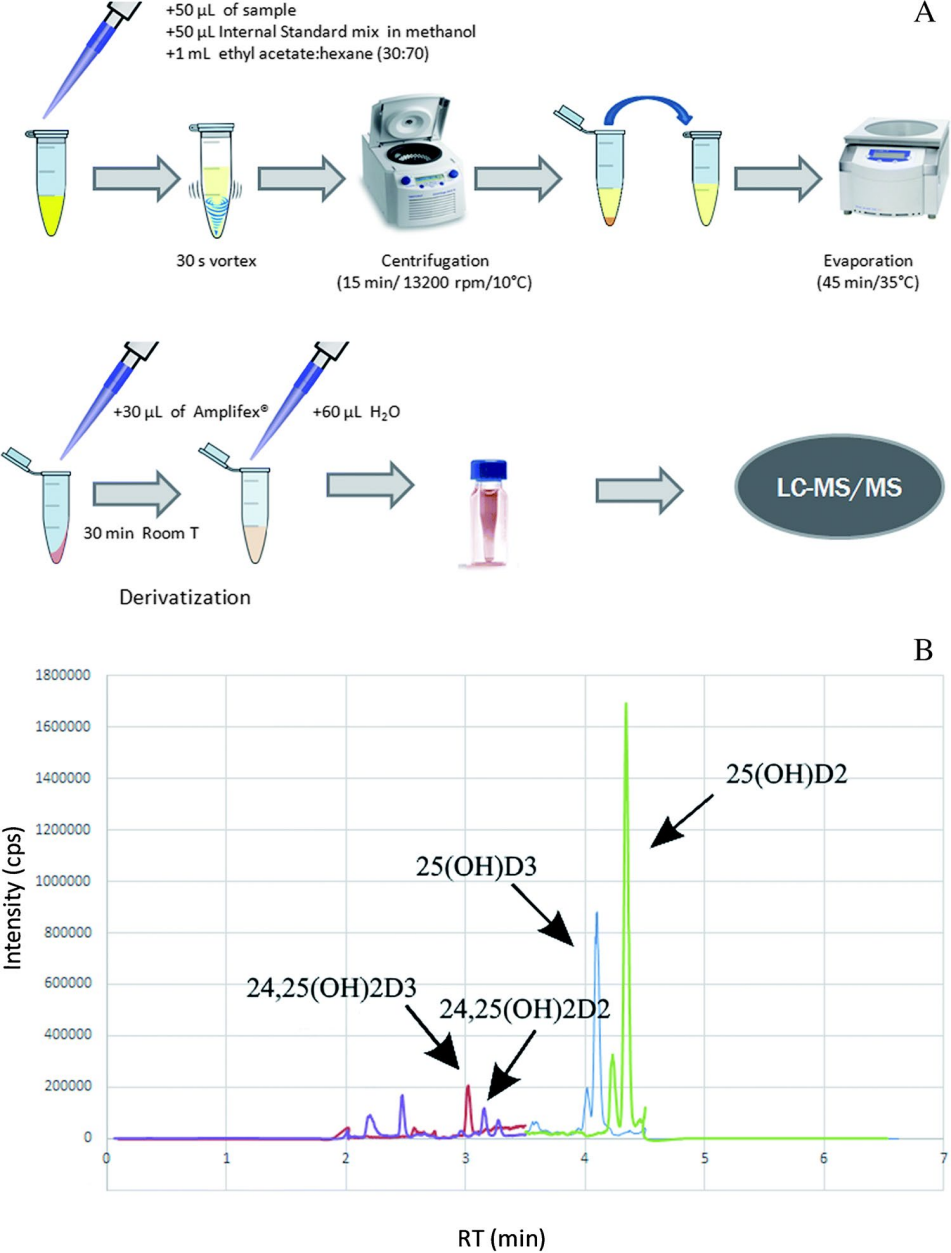
**A** Scheme of the recommended sample preparation protocol. (**B**) LC–MS/MS chromatogram with the studied vitamin D metabolites corresponding to the highest point in the calibration curve, at 5 μg/L for 25(OH)D_2_ and 25(OH)D_3_ and 0.5 μg/mL for 24,25(OH)_2_D_2_ and 24,25(OH)_2_D_3_. A C-18 column was used and the mobile phase consisted of A: water and B: acetonitrile, modified both with ammonium formate (1 mM) and formic acid (0.01%). Reprinted from ref. [70] with permission of Royal Society of Chemistry.

Rullo et al. analysed 112 samples of aqueous and vitreous humour for 25(OH)D_3_ by LC–MS/MS (70). Briefly, the protocol used 100 μL of sample and included three separate steps of LLE: 100 μL of 0.4 M ZnSO_4_, 100 μL of methanol and 2 mL of *n*-hexane. A final extraction was performed after addition of 100 μL methanol and 2 mL *n*-hexane. The supernatants were combined, evaporated to dryness and reconstituted prior to LC injection. The LOQ for 25(OH)D_3_ was 0.2 ng/mL. Another LC–MS/MS method for analysis of aqueous humour was developed by Fabregat-Cabello et al., who measured 25(OH)D_3_ and 24,25(OH)_2_D_3_ metabolites (71). The LOQs for 25(OH)D_3_ and 24,25(OH)_2_D_3_ were 0.20 ng/mL and 0.02 ng/mL, respectively. Their extraction protocol was simpler than Rullo et al.’s method since it performed only a single LLE step instead of four. The analytes were extracted from 50 μL of sample after addition of internal standards with LLE using 1 mL of ethyl acetate/hexane (30:70, v/v) (Fig. 4A). After vortexing, centrifugation and evaporation, the final step was derivatization with Amplifex reagent to enhance the sensitivity of the method (Fig. 4B).

Cho et al. highlighted the importance of measuring vitamin D status in aqueous humour in patients with ocular conditions [71]. In their research, higher concentrations of 25(OH)D were measured in aqueous humour samples from patients with diabetes than senile cataract. The aqueous humour samples’ volume was 150–200 μL and was collected from the anterior chamber. However, the sample volume required for the electrochemiluminescence assay was only 40 μL. Another study associated higher levels of 25(OH)D in aqueous humour with diabetic macular edema [72].

### Human milk

Human milk contains approximately 10–70 international units (IU)/L of vitamin D [73, 74]. The recommended intake of vitamin D for infants of up to 12 months of age is 400 IU/day (10 μg) [75]. However, assuming 50 IU/L, reaching this intake would require 8 L of breast milk per day, which is obviously impossible to achieve [76, 77]. Thus infants who are exclusively breast-fed with limited exposure to light are more prone to vitamin D deficiency. Vitamin D supplementation given to lactating women can positively affect the infant’s serum 25(OH)D levels [78]. Approximately 20% of maternal vitamin D is transferred to the child through breast milk and very little 25(OH)D passes to breast milk from the mother [79]. Human milk contains phospholipids, triglycerides, free fatty acids and other lipophilic compounds and sample preparation must assure that these interferences do not interfere with the analysis.

Kamao et al. measured fat-soluble vitamins in milk [80]. For the determination of vitamin D_2_ and D_3_, and 25(OH)D_2_ and 25(OH)D_3_, 10 mL of milk underwent saponification (20 mL of pyrogallol–ethanol (7%, w/v), 6 mL of NaCl solution (1%, w/v) and 10 mL of KOH solution (60%, w/v), 70 °C, 1 h), after adding the internal standards. After the addition of 38 mL of NaCl (1%, w/v), LLE was performed twice in a separating funnel with 30 mL of hexane:ethyl acetate (9:1, v/v). After washing, dehydration, evaporation and reconstitution steps, the extract was preconcentrated and purified using normal-phase HPLC, followed by derivatization using 4-[2-(6,7-dimethoxy-4-methyl-3-oxo-3,4-dihydroquinoxalyl)ethyl]-1,2,4-triazoline-3,5-dione (DMEQ-TAD). The recoveries for the four derivatized compounds ranged from 90.9% (± 8.8%) to 105.0% (± 4.7%).

Hollis quantified vitamin D_2_, D_3_, 25(OH)D_2_ and 25(OH)D_3_ in milk samples. The sample preparation protocol was rather complex, comparable to protocols from the 1980s [81]. Several LLE steps were performed successively to extract the compounds from the matrix and reduce the lipid content from 5 mL of milk sample, followed by one or two steps of preparative liquid chromatography.

Við Streym et al. reported an alkaline saponification procedure, followed by heptane extraction without providing experimental details [82].

Gomes et al. analysed breast milk samples to determine vitamin D sulphated analogues (vitamin D-S) using LC–ESI–MS/MS in negative ion mode [83]. Protein precipitation at room temperature was the only step of their sample preparation protocol, where 8 mL of acetonitrile were added to 4 mL of milk. The recoveries ranged from 81.1 to 102%. The LODs for D_2_-S, D_3_-S, 25(OH)D_3_-S and 25(OH)D_2_-S were 2.0, 2.8, 2.4 and 2.4 pM respectively. Vitamin D_2_-S, D_3_-S and 25(OH)D_3_-S were present in milk samples, while 25(OH)D_2_-S was not detected. Wang et al. used 1 mL of breast milk and sonicated the sample in the presence of 2 mL of acetonitrile to achieve protein precipitation [84]. LLE was performed after addition of 3 mL of methanol and 4 mL of *n-*hexane, followed by sonification, centrifugation, evaporation and reconstitution prior to 25(OH)D_3_ measurement (this information was obtained from the supplementary material of ref. [84].

Gomes et al. have also measured eight non-sulphated vitamin D metabolites (vitamin D_2_, D_3_, 25(OH)D_2_, 25(OH)D_3_, 1,25(OH)_2_D_2_, 1,25(OH)_2_D_3_, 24,25(OH)_2_D_2_ and 24,25(OH)_2_D_3_) [85]. The authors optimized the sample pre-treatment procedure by investigating the efficiency of protein precipitation and saponification. Saponification resulted in vitamin D loss even when cold saponification or addition of antioxidants was incorporated. As a result, protein precipitation using 8 mL of acetonitrile in 4 mL of milk followed by LLE (twofold) (12 mL of hexane:dichloromethane (4:1, v/v)) and PTAD derivatization formed the final sample preparation protocol. Recoveries ranged from 88.2 to 105%. All of the compounds were present in human milk. Derivatization was applied to increase the sensitivity of the method since some of the metabolites, such as 25(OH)D_2_, 1,25(OH)_2_D_2_, 1,25(OH)_2_D_3_, 24,25(OH)_2_D_2_ and 24,25(OH)_2_D_3_, are found at very low concentrations in serum and human milk.

Oberson et al. studied the effect of saponification and protein precipitation in human milk samples to quantitate vitamin D_2_, D_3_, 25(OH)D_2_ and 25(OH)D_3_ [86]. Saponification negatively affected the recovery of 25(OH)D and values did not exceed 70%. On the contrary, when protein precipitation was conducted, the recovery was 70–107% for all tested analytes. Ethanol was chosen over methanol as the optimum protein precipitant for two reasons: (1) it resulted in slightly higher recovery and (2) it is more environmentally friendly. In their final protocol, 1 mL of milk was mixed with 1 mL of ethanol and the internal standards, followed by LLE (twofold) using 2.5 mL of a mixture of *n-*hexane:ethyl acetate (9:1, v/v) and PTAD derivatization.

A fully automated sample preparation method was introduced by Gjerde et al., to measure 25(OH)D_2_, 3β-25(OH)D_3_ and 3α-25(OH)D_3_ [87]. A robotic autosampler was used to prepare the samples for analysis. The internal standards were added in 200 μL of breast milk. Subsequently, protein precipitation using 200 μL of zinc sulphate and 500 μL methanol, vortexing, centrifugation and SPE were conducted. The recoveries ranged from 72 to 103%.

Gjerde et al.’s method required only very small volumes of sample, provided high reproducibility and the fully automated procedure was simple and very fast in comparison to manual protocols. Gomes et al.’s protocol for sulphated vitamin D metabolites was equally simple [83], consisting of only a single step of protein precipitation.

The other protocols are more complex but sometimes also cover additional analytes. For example, Kamao et al.’s method targeted a broad range of fat-soluble vitamins, not just vitamin D metabolites [80]. Generally, saponification is performed when the lipids have to be removed from the sample’s lipids. As human milk has a high content in lipids, saponification is a suitable technique during sample pre-treatment. Gomes et al. [85] and Oberson et al. [86] reached the conclusion, however, that saponification has the potential to be detrimental to vitamin D analysis and thus only protein precipitation was performed, with no negative effects on the measurement. Derivatization may be required as additional sample preparation step if low abundant metabolites are to be measured.

### Hair

**Fig. 5:**
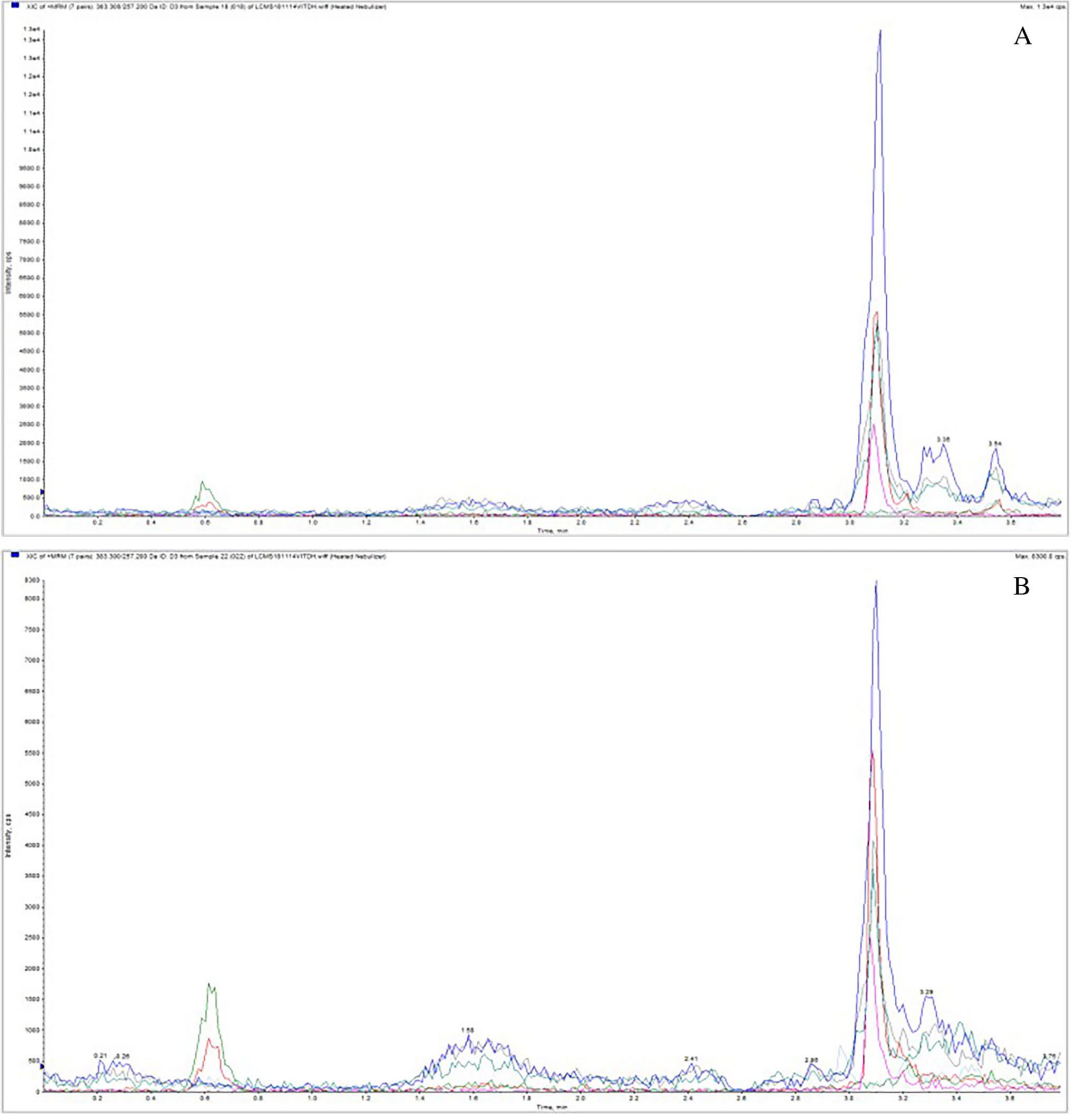
LC–MS/MS chromatograms (MRM) of (**A**) human hair and (**B**) beard. (Large blue peak represents hair extracted 25(OH)D_3_. Red peak is the assay 25(OH)D_3_ internal standard.) from ref. [88] with permission under the CC BY 4.0 license

The presence of 25(OH)D_3_ has also been shown in hair. This was demonstrated for the first time by Zgaga et al. using LC–MS/MS [88]. Washing was required prior to the main sample preparation steps, to remove external contamination, hair products, sweat and sebum. Without careful design of this washing step, analyte loss occurred, resulting in underestimation of the analyte’s concentration. In Zgaga et al.’s study, 20–44 mg of hair was used and a single step of washing was conducted using 2 mL of isopropanol (vortex, 2 min, room temperature). Subsequently, extraction with 1.4 mL of methanol was performed (with mixing for 18 h), followed by evaporation and reconstitution. A LC–MS/MS chromatogram of a hair sample is given in Fig. 5A, while Fig. 5B shows a beard sample. The average concentration in hair was 421 pg/mg (range from 26.5 to 911 pg/mg) for a single subject, and for the beard sample, it was 231 pg/mg.

The second method in the literature was presented by Shah et al., who washed 500 mg of hair using a mixture of methanol:water and then analysed 200 mg of the cleaned hair, which was previously pulverized [89]. As the more polar nature of the washing solvent could potentially result in extraction of the analytes of interest, the washing solutions were analysed after the washing step. No extraction was observed, however. Analyte extraction using 2 mL of methanol:water (1:1, v/v) (sonification, 2 h), filtration through a PTFE membrane (0.45 μm), evaporation and reconstitution were performed for sample preparation of 25(OH)D, which was then determined at pg/mg levels, similar to the observed concentrations by Shah et al. [88].

Comparing the two methods, it is noteworthy that Zgaga et al. used a less polar solvent than Shah et al. for the wash step to avoid extraction of 25(OH)D_3_. Another difference is the amount of sample used, which was lower in Zgaga et al.’s assay. This is potentially important as in some cases limited hair amounts may be available, for example from new-borns or elderly subjects, which may not have sufficient scalp hair. Shah et al. used a slightly larger amount (200 mg) as suggested by Society of Hair Testing (SoHT) for exogenous substance analysis (10–50 mg of hair [90]. The SoHT also recommends pulverizing the sample after washing to ensure sample’s homogeneity [90], as performed by Shah et al., which can lead to higher extraction efficiency [91].

### Saliva

**Fig. 6:**
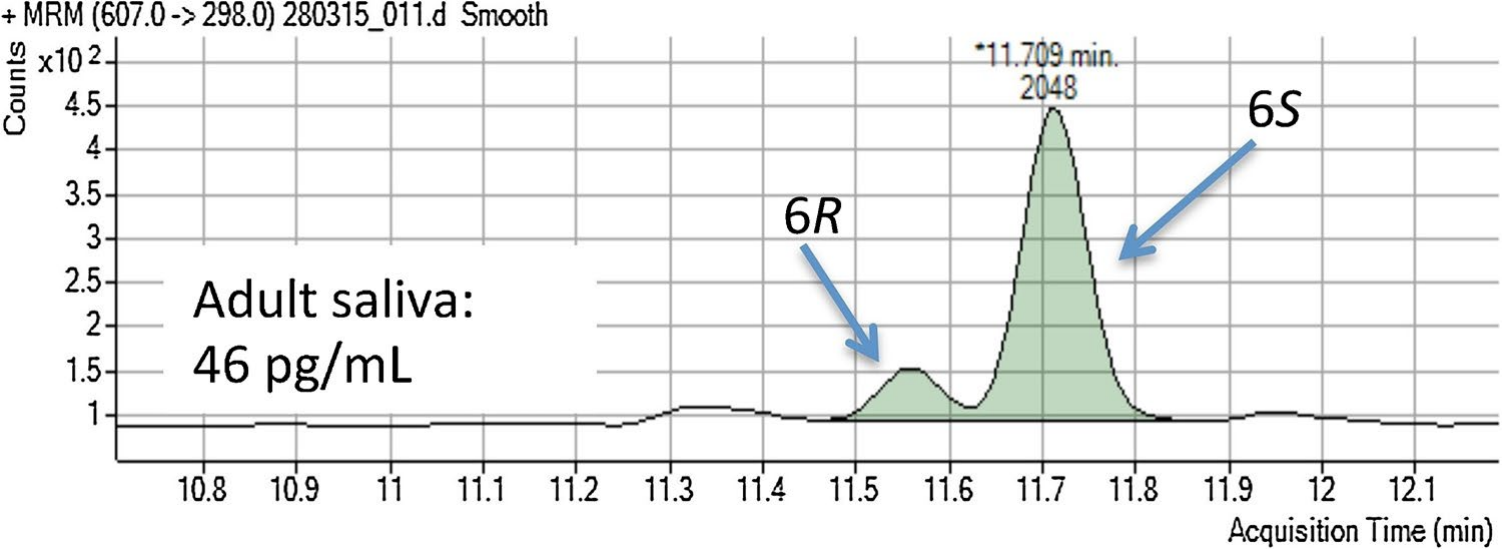
LC–MS/MS chromatogram of 25(OH)D_3_’s 6S- and 6R-isomers produced by PTAD derivatization of an adult human saliva sample (concentration of 25(OH)D_3_, 46 pg/mL). Reprinted from ref. [94] with permission from Elsevier

The presence of vitamin D species in human saliva has been frequently investigated since the 1980s. Fairney et al. measured 25(OH)D in saliva samples using a competitive protein-binding assay after a sample preparation protocol requiring multiple steps [92]. The measured concentration ranged from 105 to 1000 pg/mL, with values varying throughout the day. Higashi et al. determined 25(OH)D_3_ in saliva samples using LC–MS/MS [93]. The sampling method they used was unstimulated and did not include any device to avoid non-quantitative recovery and contamination. The samples were centrifuged to remove debris, cells and mucous, and stored at − 20 °C. After addition of the internal standard, 2 mL of acetonitrile was added to 1 mL of sample to achieve protein precipitation. SPE, evaporation and PTAD derivatization concluded the sample preparation. The group observed a good correlation between serum and saliva levels, with levels of 25(OH)D_3_ in saliva approx. 1000-fold lower than those in serum. Moreover, the concentrations measured in this study (3–14.8 pg/mL) were lower than those observed by Fairney et al. [92] A similar sample preparation method was presented by Clarke et al., where 1 mL of saliva underwent LLE with 3 mL of ethyl acetate, followed by PTAD derivatization prior to LC–MS/MS analysis (Fig. 6) [94]. Clark et al. suggested that the proposed SPE method, which was based on Higashi et al.’s [93] protocol, was more reliable than LLE even if it was more time consuming.

ELISA has also been used to determine 25(OH)D_3_ in saliva. By employing this technique, salivary 25(OH)D_3_ levels of menopausal women with xerostomia were found to be higher than those in the control group [95]. In a different study, salivary 25(OH)D_3_ levels were lower in patients with oral lichen planus than in the control group [96].

Salivary flow rate, pH, contamination, oral trauma, sampling conditions and other factors can affect the concentration of a compound. The sampling procedure in this particular matrix plays a crucial role in the accuracy of the results. A chewing gum is often employed as it can increase the production of saliva and minimize the sampling time. Higashi et al. observed an overestimation in salivary 25(OH)D_3_ when the sampling was performed with the help of chewing gum [97]. A thorough investigation concerning the challenges and factors that can affect vitamin D measurement in saliva samples was conducted by Clarke et al. [94]. In order to develop and optimize their assay, the authors examined the effects of (1) stimulated versus passive collection method, (2) sequential sampling and storage temperature, (3) mucin removal and (4) time of day collection and flow rate. Passive collection resulted in higher 25(OH)D_3_ levels as compared to stimulated collection by chewing a synthetic swab [94]. Moreover, vitamin D-binding protein and albumin levels were higher when passive collection was used. Freezing samples immediately or after 24 h or refrigeration had no effect. Lower concentrations were observed when samples were collected 10 min after awakening as compared to those collected immediately after awakening. Time of the day had a little impact on 25(OH)D_3_ concentration. Mucin seemed to interfere in 25(OH)D_3_ measurement; thus, centrifugation steps to remove it were necessary [94].

### Urine

Urine as biological matrix offers many advantages over other biological fluids. Urine is available in large amounts and sampling is very easy in a non-invasive manner. The analysis of urine samples for vitamin D profiles has the potential to reveal more information on the metabolism of vitamin D, in particular concerning vitamin D excretion [98].

**Fig. 7:**
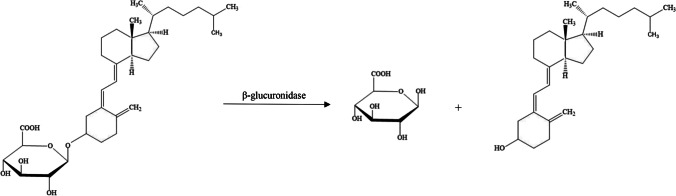
β-Glucuronidase hydrolysis reaction for vitamin D_3_ glucuronide

Hydrolysis using β-glucuronidase is a common first step in urine pre-treatment for chromatography, as the highly polar glucuronide metabolites are not easily retained by commonly applied reversed-phase LC columns. Hydrolysis of glucuronidated metabolites reverts these molecules back to their ‘parent’ compounds [99]. The β-glucuronidase hydrolysis reaction for vitamin D_3_-glucuronide is presented in Fig. 7. As injecting enzymes onto the column can dramatically decrease lifetime of the stationary phases, methods usually include a dilution step of the pre-treated sample or protein precipitation to reduce the concentration of the enzyme after the reaction.

Yu et al. analysed urine samples for 25(OH)D_3_, 1,25(OH)D_3_ and the intact vitamin D_3_-sulphate conjugate [100]. After sampling, urine was filtered twice through a filter paper and 0.45-μm Nylon filter and stored at − 20 °C. Reversed-phase SPE (C-18) was the chosen sample preparation method for 3 mL of urine.

Higashi et al.’s LC–MS/MS method revealed the presence of 23,25(OH)_2_D_3_, 24,25(OH)_2_D_3_ and 23,25(OH)_2_–24-oxo-D_3_ as glucuronides in human urine under physiological conditions [98]. According to the authors, they were the first to report 23,25(OH)_2_–24-oxo-D_3_ in human fluids. The group suggested that C-23 hydroxylation is an important mechanism for vitamin D_3_ excretion. Their sample preparation protocol started with a urine sample of 2 mL, which was incubated with β-glucuronidase in 0.1 M sodium acetate-acetic acid buffer (pH 5, 37 °C, 2 h). The internal standard and 2 mL of acetonitrile were added to the reaction mixture. After centrifugation, the supernatant underwent reversed-phase SPE (OASIS HLB) and the eluent from the first SPE step underwent further normal-phase SPE (Bond Elut SI). The last step of sample preparation was derivatization with DMEQ-TAD; a Cookson-type reagent.

**Fig. 8:**
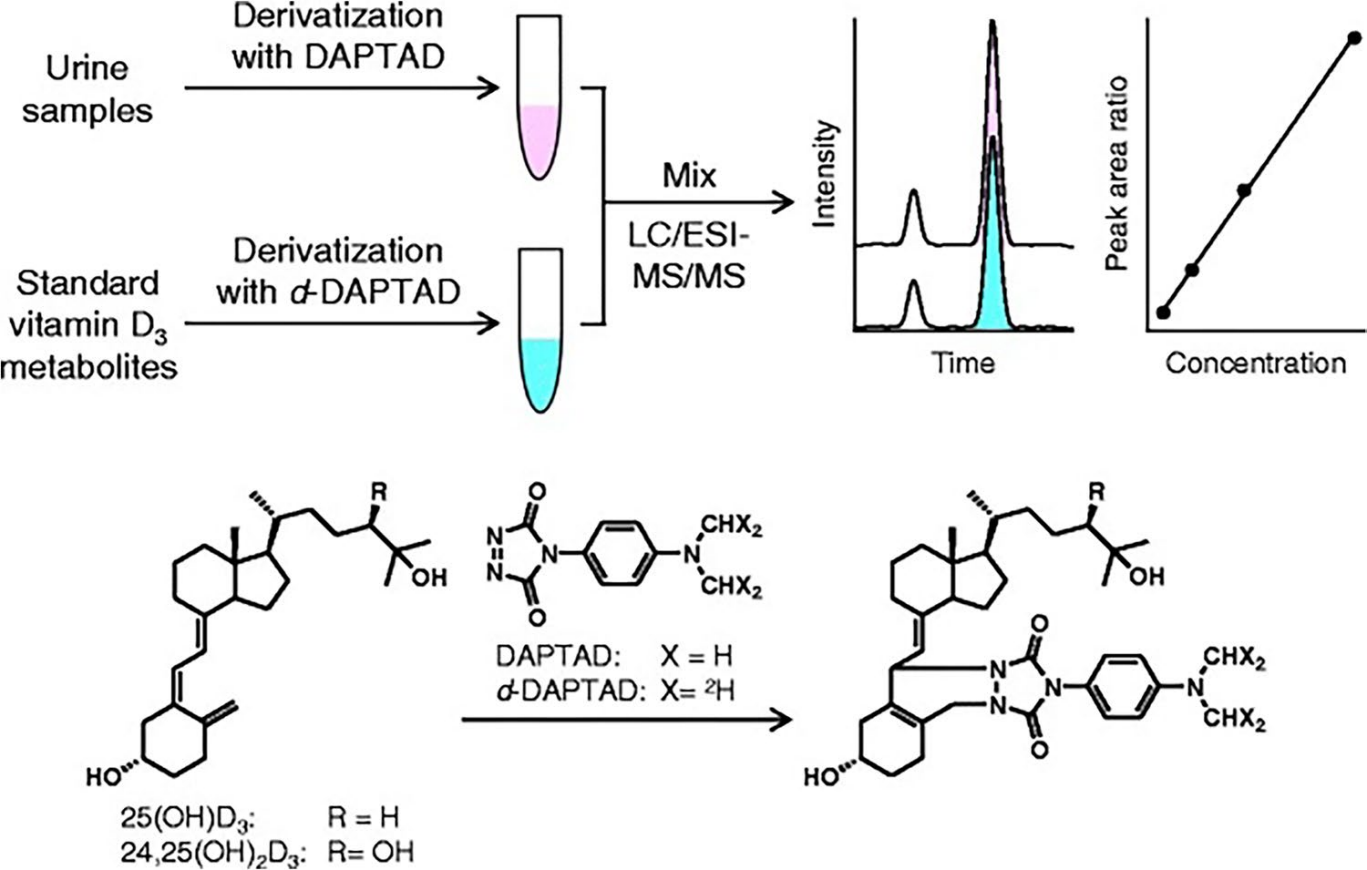
Scheme of quantification procedure of vitamin D_3_ metabolites in urine based on electrospray ionization enhancing and isotope-coded derivatization. Reprinted from ref. (102) with permission from Springer Nature (*Analytical and Bioanalytical Chemistry*)

Ogawa et al. detected 25(OH)D_3_ and 24,25(OH)_2_D_3_ in urine using LC–MS/MS [101]. Their approach was very innovative as instead of using a stable isotope-labelled analogue of each analyte, they introduced a stable isotope-coded derivatized moiety as shown in Fig. 8. The sample preparation approach has some similarity to the procedure described by Higashi et al.: a urine sample of 1 mL was incubated with β-glucuronidase in 0.1 M sodium acetate-acetic acid buffer (pH 5, 37 °C, 2 h). Subsequently, 1 mL of acetonitrile was added to the reaction mixture and, after centrifugation, the supernatant was extracted using reversed-phase SPE (OASIS HLB). The dry residue of the sample was derivatized with 4-(4′-dimethyl amino phenyl)-1,2,4-triazoline-3,5-dione (DAPTAD). Prior to analysis, the isotope-labelled analyte standards were added to the sample.

**Fig. 9:**
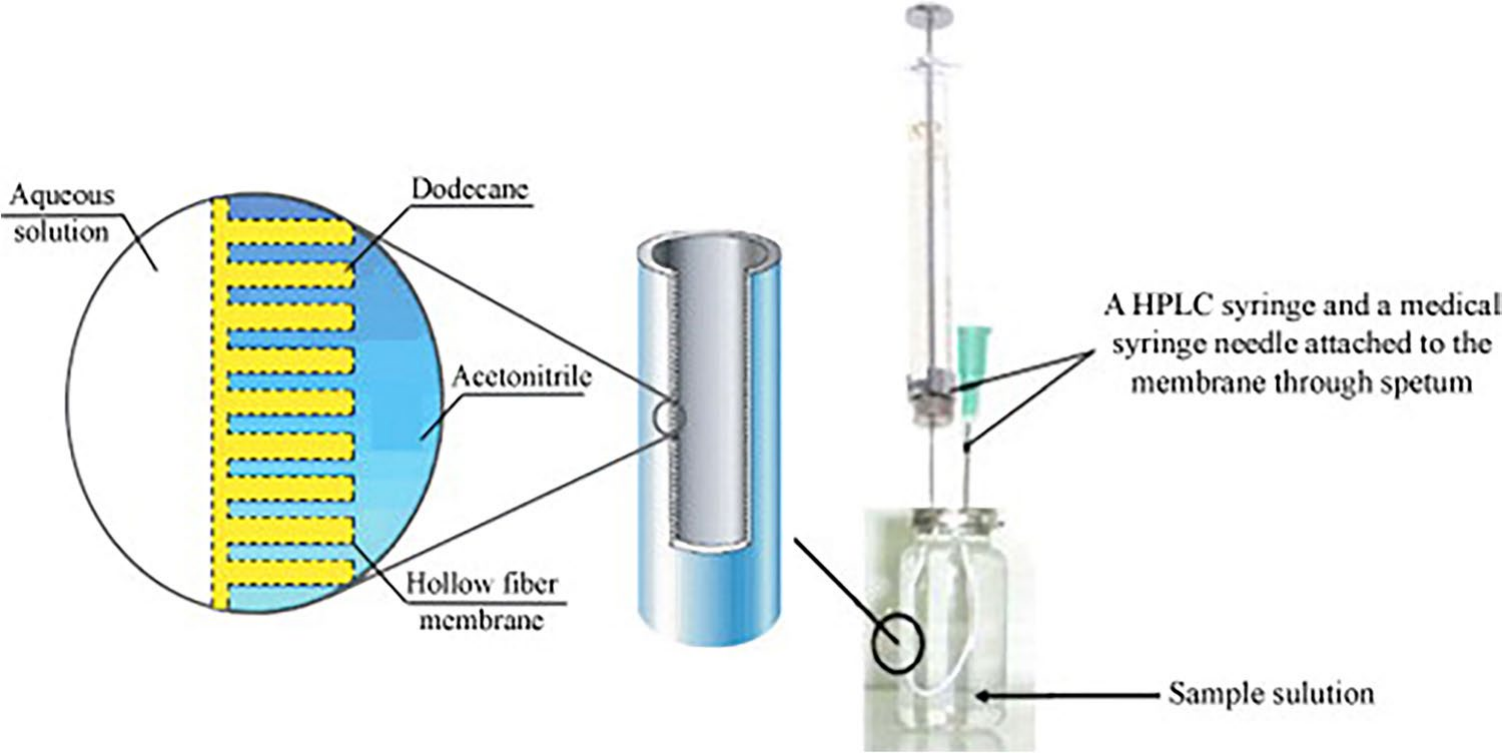
Schematic diagram of a three-phase HF-LPME based on two immiscible organic solvents. Reprinted from ref. [103] with permission from Springer Nature (*Microchimica Acta*)

An alternative approach was proposed by Saber-Tehrani et al., who developed a hollow fibre liquid-phase microextraction (HF-LPME) method for the extraction of vitamin D_3_ from plasma and urine (103). In HF-LPME, 15–20 μL of an extracting solvent is immobilized on the fibre wall forming a supported liquid membrane (SLM) (104). Moreover, liquid solvent (either the same solvent as SLM or a different one) is placed in the lumen of the hollow fibre. Analytes from an aqueous sample are extracted into the solvent forming the SLM and into the acceptor solution located inside the lumen of the hollow fibre. HF-LPME can be characterized as a two-phased or three-phased process. In two-phase HF-LPME, the solvent forming the SLM and the accepting solvent are the same. If the two solvents are different, either immiscible organic solvents or organic/aqueous solvents, the HF-LPME is characterized as three-phased process. The acceptor solution is removed by a micro syringe prior to analysis. HF-LPME offers the advantage of simultaneous sample preconcentration and purification. Figure 9 gives an example of a three-phase HF-LPME in a U-shaped configuration.

In Saber-Tehrani et al.’s application, the urine samples were stored at − 20 °C and then filtered through a cellulose acetate membrane, followed by dilution with ultrapure water (1:1, v/v) prior to HF-LPME [102]. The U-shaped hollow fibre was placed into the sample’s vial including 8 mL of sample solution and the extraction took place at room temperature for 35 min. Finally, the acceptor phase was withdrawn into the syringe and injected into the LC-UV system. The authors performed a three-phase HF-LPME, where the urine sample, *n*-dodecane as SLM and acetonitrile as accepting solvent form the three-phase system. *N*-dodecane’s immobilization was based on diffusion and the extraction of the sample’s analytes between the two organic solvents was based on the concentration gradient between the two immiscible solvents. The authors optimized several important parameters: (1) selection of organic solvent forming the SLM, (2) length of the hollow fibre, (3) stirring speed, (4) extraction time, (5) salt addition, (6) sample volume and (7) extraction temperature. The recovery of the method was > 93% for two concentration levels. The final sample preparation protocol was simple, low-cost and environmentally friendly with good enrichment factors (= 121) and recovery.

### Soft tissues other than adipose tissue

Beumer et al. measured 1,25(OH)_2_D_3_ in mice tumours after intraperitoneal injection of the compound [104]. Tumours were excised, frozen in liquid nitrogen and stored in − 80 °C. The tumours were homogenized in phosphate buffer saline (1:3, w/v) prior to use. The internal standard and 1 mL of methylene chloride were added to 200 μL of tumour homogenate and LLE was performed. The bottom layer underwent SPE on an amino phase, followed by evaporation and reconstitution.

Beef, pork, lamb and chicken meat were analysed by Strobel et al. to determine vitamin D_2_, D_3_, 25(OH)D_2_ and 25(OH)D_3_ using LC-APCI-MS/MS (106). Meat is a complex biological matrix, since it contains many endogenous and exogenous compounds that can give interferences; thus, sample preparation usually requires multiple steps. Antioxidant sodium ascorbate (0.5 g) was added to 7.5 g of meat sample. For saponification, 10 mL of deionized water, 25 mL of ethanol and 7.5 g of KOH were added (shaking, 25 °C, 15 h). The saponification extract was passed through a SPE cartridge filled with diatomaceous earth, followed by centrifugation, evaporation, reconstitution and filtration (0.2 μm) to achieve the optimum purification prior to LC injection. Higher concentrations of vitamin D_3_ and 25(OH)D_3_ were observed in pork and chicken meat. The concentration of vitamin D_3_ ranged from 0.06 to 0.18 μg/100 g and for 25(OH)D_3_ from 0.05 to 0.21 μg/100 g. A similar sample preparation method for the determination of vitamin D_3_ and 25(OH)D_3_ in pork meat and lard was presented previously by Jakobsen et al. and Clausen et al. using HPLC–DAD [106, 107]. In their protocol, the authors used 50 g of meat and 10 g of lard, which were saponified followed by LLE with petroleum ether and SPE (silica). A second clean-up step involved a semi-preparative HPLC system with an amino column connected to a silica column.

Lipkie et al. analysed soft tissues (liver, epididymal fat, gastrocnemius muscle) from Spargue-Dawley rats using LC–ESI–MS/MS [108]. Tissues were homogenized in phosphate buffer saline and stored at − 80 °C. Protein precipitation was achieved by addition of 400 μL acetonitrile to 400 μL of the homogenate. LLE (400 μL, methyl tert-butyl ether) and SPE (reversed-phase) followed prior to PTAD derivatization.

The sample preparation protocols summarized in this section cannot be directly compared as their aims were quite different, that is either food analysis, where sample amounts of sample are usually available, or tissue analysis in biomedical applications, where the amounts of available material may be very limited (tumour, rat soft tissues). That is, method development objectives were naturally very different. Interestingly, saponification was performed in virtually all protocols as part of the sample pre-treatment, despite its time consuming nature, because of the need to remove interfering lipids [109].

### Synovial fluid

Synovial fluid is found at the joints of the knees, shoulders, hips, hands and feet. It is a combination of filtered plasma that enters the joint space and hyaluronic acid [110]. It has two functions: (1) it acts a lubricant for joint surfaces and (2) it is a source of nourishment.

Li et al. analysed synovial fluid samples from patients with rheumatoid arthritis using LC–MS/MS (112), with the aim of comparing the concentration levels of vitamin D metabolites in synovial fluid and serum. Proteins were first removed by protein precipitation, analytes extracted by supported liquid extraction (SLE), followed by derivatization using DMEQ-TAD. While SLE shares some similarities with SPE, in SLE, the inert material retains the entire sample and only the analytes of interested are eluted. SLE requires fewer steps than SPE; it is essentially only ‘load-wait-elute’. The authors’ protocol started with pre-treatment of 220 μL of sample with 80 μL of methanol, 50 μL isopropanol and 80 μL of water to achieve protein precipitation [19]. After removal of the proteins, the remaining supernatant was transferred onto the SLE plate. The sample was completely absorbed by the SLE sorbent by applying vacuum. After 6 min, the analytes of interest were eluted using 2 × 800 μL of MTBE/ethyl acetate (90/10), initially under gravitational force, followed by application of vacuum. Subsequently, the eluent was evaporated under nitrogen and reconstituted. The authors observed that the levels of vitamin D metabolites in synovial fluid were lower than those in the corresponding serum samples. The authors point out the utility of analysing samples beyond serum, since the vitamin D status measured from circulating vitamin D levels provides limited perspective on the extra-skeletal actions of vitamin D. In pathophysiological processes such as rheumatoid arthritis, the localized concentration of 25(OH)D_3_ may be responsible for the anti-inflammatory action of vitamin D [112].

## Conclusions

The present review summarizes important non-conventional biological matrices with respect to their suitability as sample matrix for analysis of vitamin D metabolites, for obtaining comparable or complementary information to that from analysis of serum or plasma.

Table 1 summarizes the different biological matrices, from which vitamin D compounds have been measured, the range of required amounts of sample, the most commonly applied steps during sample preparation, and typical concentration levels found in these matrices. Homogenization is the first step for most non-liquid samples, frequently followed by protein precipitation or saponification. The dominant extraction techniques are LLE and SPE, which in recent years have considerably improved in terms of efficiency. Often preparative liquid chromatography has been replaced by more state-of-the-art sample preparation methods, which are simpler to use and require smaller sample volumes/weights. Recent instrumental developments of sample preparation techniques (e.g. efficient, small-scale SPE in well plate format) have enabled faster and less complicated sample pre-treatment procedures, usually followed by state-of-the-art LC–MS/MS. Matrix interferences can be detrimental for LC–MS, since ionization suppression negatively influences the accuracy and sensitivity of the method [113]. Derivatization as final step of the sample preparation method is often employed to overcome ion suppression problems and enhance sensitivity.

**Table 1:** Non-conventional biological matrices for determination of vitamin D metabolites

Sample/Ref	Sample volume/weight in reviewed studies	Sample preparation steps	Measured vitamin D compounds	Observed concentrations on the order of
Adipose tissue [37–40]	0.2–2 g	1. Homogenization	D_3_	ng/g
		2. Saponification	D_2_	
		3. LLE	25(OH)D_3_	
		4. SPE		
Amniotic fluid [43–45]	60 μL–25 mL	1. LLE	25(OH)D_3_	pg/mL to ng/mL
		2. Further purification using LC or	1,25(OH)_2_D_3_	
			24,25(OH)_2_D_3_	
		1. Protein precipitation		
Human brain tissue [53]	0.1 g	1. Homogenization	D_3_	pg/g
		2. LLE	25(OH)D_3_	
			1,25(OH)_2_D_3_	
			24,25(OH)_2_D_3_	
CSF [46, 49]	200–400 μL	1. Protein precipitation	25(OH)D_3_	ng/mL
			24,25(OH)_2_D_3_	
Aqueous or Vitreous humour [69–71]	40–100 μL	1. LLE	25(OH)D_3_	ng/mL
			24,25(OH)_2_D_3_	
Tear fluid [60]	35 μL from each eye	1. Extraction	25(OH)D_3_	ng/mL
Human milk [80–87]	200 μL–10 mL	1. Protein precipitation or	D_3_	ng/mL
			D_2_	
		1. Saponification	25(OH)D_2_	
		2. LLE or SPE	25(OH)D_3_	
		3. Further purification using LC	3α-25(OH)D_3_	
			D_2_-S	
			D_3_-S	
			25(OH)D_3_-S	
Hair [88, 89]	20–200 mg	1. Washing	25(OH)D_3_	pg/mL
		2. Extraction		
Saliva [92, 93]	1 mL	1. Protein precipitation	25(OH)D_3_	pg/mL
		2. LLE or SPE		
Urine [98, 100–102]	1–4 mL	1. Filtration	D_3_-sulphate	pg/mL
		2. Enzymatic hydrolyzation		
			25(OH)D_3_	
		3. SPE or	1,25(OH)_2_D_3_	
		2. HF-LPME	23,25(OH)_2_D_3_	
			24,25(OH)_2_D_3_	
			23,25(OH)_2_–24-oxo-D_3_	
Soft tissues [104–108]	7.5–50 g	1. Homogenization	D_3_	μg/100 g
		2. Saponification or protein precipitation	25(OH)D_3_	
		3. LLE		
		4. SPE		
		5. Further purification using LC		
Synovial fluid [19, 111]	220 μL	1. Protein precipitation	25(OH)D_2_	ng/mL except for 1,25(OH)_2_D_3_ (pg/mL)
			25(OH)D_3_	
			3α-25(OH)D_3_	
		2. SLE	24,25(OH)_2_D_3_	
			1,25(OH)_2_D_3_	

It is clear that none of the described alternative matrices currently has the potential to replace blood-based matrices as a universal sample type for vitamin D analysis, e.g. for status marker measurements or for broad metabolite profiling applications. Importantly, in some of the applications reviewed in this article, the analysis of the non-conventional sample was accompanied by analysis of serum or plasma and the comparison of metabolite concentrations between these compartments can potentially provide additional insight on the role of vitamin D in specific health or disease questions, where blood levels may remain unaffected. In addition, some alternative sample matrices allow non-invasive sampling, permit easy shipment of samples and only require simple storage at ambient conditions.

In conclusion, we hope that the present review is useful to researchers, to give them an overview of the different biological samples for vitamin D analysis and the required sample preparation steps to isolate vitamin D metabolites from these sample matrices for subsequent instrumental analysis, which today is usually conducted by LC–MS.
